# KL-6 Mucin as Serum Tumor Marker of Metastatic Renal Cancer: A Case Report

**DOI:** 10.1155/2024/6648459

**Published:** 2024-09-27

**Authors:** Kyohei Ishida, Go Hasegawa, Toshinori Takada, Akira Ogose, Gen Kawaguchi, Yohei Ikeda, Hiroki Nishiyama, Noboru Hara, Tsutomu Nishiyama

**Affiliations:** ^1^ Department of Urology Uonuma Institute of Community Medicine Niigata University Medical and Dental Hospital, Minamiuonuma, Niigata, Japan; ^2^ Department of Pathology Uonuma Institute of Community Medicine Niigata University Medical and Dental Hospital, Minamiuonuma, Niigata, Japan; ^3^ Department of Respiratory Medicine Uonuma Institute of Community Medicine Niigata University Medical and Dental Hospital, Minamiuonuma, Niigata, Japan; ^4^ Department of Orthopedic Surgery Uonuma Institute of Community Medicine Niigata University Medical and Dental Hospital, Minamiuonuma, Niigata, Japan; ^5^ Department of Radiation Oncology Uonuma Institute of Community Medicine Niigata University Medical and Dental Hospital, Minamiuonuma, Niigata, Japan; ^6^ Department of Diagnostic Radiology Uonuma Institute of Community Medicine Niigata University Medical and Dental Hospital, Minamiuonuma, Niigata, Japan

**Keywords:** multiple metastases, renal cystic cancer, serum KL-6, serum tumor marker

## Abstract

We encountered a case of metastatic renal cell carcinoma in which the serum level of KL-6, a therapeutic marker, was exceptionally high and fluctuated with the progression of treatment. A 74-year-old man was diagnosed with right renal cystic cancer and multiple metastases in October 2022. The KL-6 level was 27490 U/mL. He started treatment with lenvatinib and pembrolizumab. KL-6 decreased to 3885 U/mg in February 2023. The patient's proteinuria worsened, leading to the discontinuation of lenvatinib. KL-6 increased to 25950 U/mL in April. He discontinued pembrolizumab and started taking cabozantinib. In September, drug-induced bilateral inflammatory pneumonitis developed. He discontinued cabozantinb and began taking axitinib. KL-6 decreased; however, he suffered from severe diarrhea and subsequent renal insufficiency. He discontinued axitinib in November. KL-6 increased to 29640 U/mL in December.

## 1. Introduction

Blood levels of the high-molecular-weight glycoprotein Krebs von den Lungen-6 (KL-6) increase when interstitial pneumonia, a lung disease, develops [1, 2]. Therefore, it is often used as an indicator to diagnose and evaluate the activity of interstitial pneumonia.

We encountered a case of metastatic renal cell carcinoma with extremely high serum KL-6 levels, which may be a therapeutic marker.

## 2. Case Presentation

**Figure 1:**
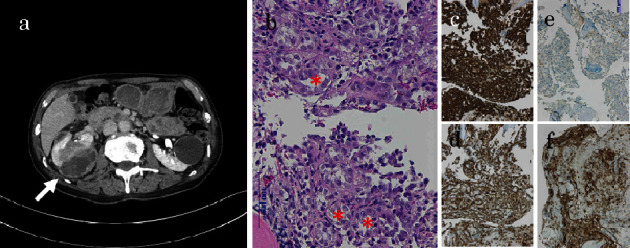
Computed tomography (a) revealed a right cystic renal tumor (arrow). Hematoxylin and eosin staining (b) showed a tumor with lumen (asterisk) along with bone tissue. The tumor was positive for CAM5.2 (c) and vimentin (membrane) (d), and weakly positive for CD10 (e), indicating bone metastasis of renal cell carcinoma. The tumor was positive for MUC1 (f) and produced KL-6.

**Figure 2:**
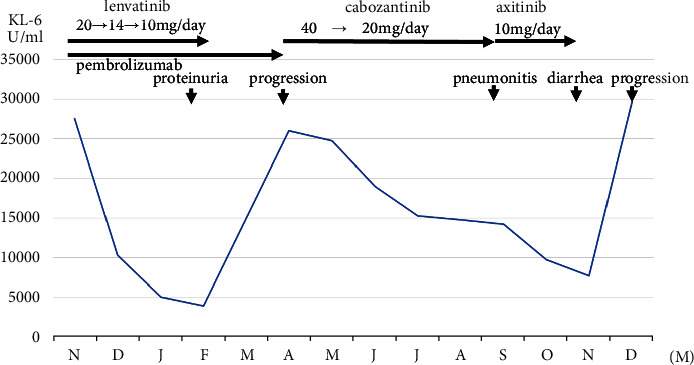
Serum KL-6 levels fluctuated depending on the patient's disease status related to treatment.

A 74-year-old man visited our hospital complaining of back pain in October 2022. Computed tomography (CT) revealed a cystic tumor in the right kidney, suspected to be cystic renal cancer with multiple bone, liver, and right adrenal metastases (Figure 1). He underwent vertebral stabilization surgery (Th10-12). Pathological findings revealed bone metastasis from renal cell carcinoma in the metastatic bone lesion (Figure 1). We did not perform a renal tumor biopsy because it was a cystic tumor. He was diagnosed with right renal cystic cancer and multiple metastases. He underwent palliative radiation (30 Gy/10 fr) at the vertebral level (Th9-L2). He decided to start treatment with lenvatinib (20 mg/day) and pembrolizumab (200 mg once every 3 weeks), and KL-6 was measured as part of the pretreatment evaluation. The KL-6 value was abnormally high at 27490 U/mL (Figure 2). Following treatment with lenvatinib and pembrolizumab, KL-6 decreased to 3885 U/mg in February 2023; however, he suffered from hypothyroidism, thrombocytopenia (Grade 2), and proteinuria. The dose of lenvatinib was reduced from 20 to 14 mg, and then to 10 mg. However, the patient's proteinuria worsened, leading to the discontinuation of lenvatinib in February 2023. CT in February 2023 revealed that the tumor in the right renal tumor had decreased in size. Pembrolizumab was continued. CT in April 2023 revealed that the right renal tumor had slightly increased in size, and the KL-6 levels had elevated to 25950 U/mL (Figure 2). He discontinued pembrolizumab and started taking cabozantinib at a dose of 40 mg/day in April 2023. The KL-6 level gradually decreased, but proteinuria was observed. As a result, the dose of cabozantinib was reduced to 20 mg/day in May 2023. CT in September 2023 revealed that bilateral inflammatory pneumonitis had developed as a result of drug-induced reaction. He discontinued cabozantinb and started taking axitinib at a dose of 10 mg/day in September 2023. The KL-6 level then decreased; however, he suffered from severe diarrhea and subsequent renal insufficiency. He discontinued axitinib and transfusion therapy in November 2023. CT in December 2023 revealed that the right renal tumor and metastatic lesion in the liver had slightly increased in size. The KL-6 level increased to 29640 U/mL (Figure 2). He resumed taking axitinib at a dose of 6 mg/day in January 2024, after his diarrhea had improved. However, the patient's condition suddenly deteriorated, and he died of progression of renal cell carcinoma in February 2024.

## 3. Discussion

The transmembrane glycoprotein mucin 1 (MUC1), also known as KL-6, is a macromolecular protein. It is also referred to as MUC1, EMA, MCD, PEM, PUM, or MAM6. Owing to its structural and biochemical properties, KL-6 can act as a lubricant, moisturizer, and physical barrier in normal cells. It is a substance produced by Type II alveolar epithelial cells in the lungs, is now classified as a human MUC1 mucin protein, and regenerating Type II pneumocytes are the primary cellular source of KL-6/MUC1 in the affected lungs of patients with interstitial lung diseases (ILDs) [1, 2]. KL-6 levels in the blood increase when interstitial pneumonia, a lung disease, develops. Therefore, it is often used as an indicator to diagnose and evaluate the activity of interstitial pneumonia.

In cancer cells, MUC1 often undergoes aberrant glycosylation and overexpression. It is involved in cancer invasion, metastasis, angiogenesis, and apoptosis due to its participation in intracellular signaling processes and the regulation of related biomolecules [3]. KL-6 is also considered a potential marker of renal tumors [4]. Fukushima et al. demonstrated that cytoplasmic expression of KL-6 antigen is a defining characteristic of chromophobe renal cell carcinoma [4]. In the present case, a biopsy could not be obtained from the primary lesion due to cystic renal cell carcinoma. The pathological tissue from the fracture site tested positive for CAM5.2 and vimentin (membrane) and exhibited weak positivity for CD10, leading to a diagnosis of metastasis from renal cell carcinoma. It was concluded that diagnosing the malignant phenotype from this pathological specimen would be challenging; however, based on reports of KL-6 antigen overexpression in renal tumors, particularly in chromophobe renal cell carcinoma, the current case may be chromophobe renal cell carcinoma [4]. Since the patient in this case had multiple bone metastases, liver metastasis, and adrenal metastasis at the time of diagnosis, the clinical malignancy is considered to be high. The underlying reason for this phenomenon remains unknown; however, aberrant expression of KL-6/MUC1 mucin has been proven to be associated with poorer tumor behavior in many carcinomas and the aberrant expression of KL-6/MUC1 mucin may be closely associated with the malignant potential of the present case [5–7].

Although there have been reports of elevated serum KL-6 antigen concentrations in patients with malignant tumors, including lung cancer, breast cancer, pancreatic cancer, and hepatocellular carcinoma, no reports have indicated elevated serum KL-6 concentrations in patients with renal cell carcinoma [8, 9]. Serum KL-6 may be valuable in clinical practice as a tumor marker of various cancers. It has been suggested to play a crucial role, especially in monitoring disease recurrence. Overexpression of KL-6 antigen in renal tumors, particularly in chromophobe renal cell carcinoma, may lead to increased serum KL-6 concentrations in patients with renal cell carcinoma [4]. This suggests that KL-6 antigen may serve as a potential tumor marker for renal carcinoma, as demonstrated in the present case. Serum KL-6 levels will be measured to identify any adverse events associated with treatments, such as immune checkpoint inhibitors. However, there have been no reports indicating that serum KL-6 is useful as a tumor marker for renal cell carcinoma to date. In the future, there may be additional reports of cases in which serum KL-6 could be utilized as a tumor marker to monitor treatment progress in renal cell carcinoma, as demonstrated in the present case.

## 4. Conclusion

We encountered a case of metastatic renal cell carcinoma in which the serum level of KL-6, a therapeutic marker, was exceptionally high and fluctuated with the progression of treatment.

## Data Availability

The data that support the findings of this study are available from the corresponding author upon reasonable request.
